# Microbial contamination of donor corneas and post-keratoplasty endophthalmitis: a comparison between Japanese and U.S. eye banks using cold storage

**DOI:** 10.1038/s41598-026-60897-w

**Published:** 2026-07-03

**Authors:** Naoya Nakagawa, Yuto Yukari, Ami Igarashi, Masato Takeda, Yuki Mizuki, Hiroyuki Nakashizuka, Satoru Yamagami, Takahiko Hayashi

**Affiliations:** 1 Department of Ophthalmology, Department of Visual Sciences, Nihon University School of Medicine, Tokyo, Japan; 2 Department of Ophthalmology, Yokohama Minami Kyosai Hospital, Yokohama, Japan; 3 Department of Ophthalmology and Visual Science, Yokohama City University Graduate School of Medicine, Yokohama, Japan; 4 Division of Ophthalmology, Department of Visual Sciences, Nihon University School of Medicine, Nihon University Hospital, Tokyo, Japan; 5 Division of Ophthalmology, Department of Visual Sciences, Nihon University School of Medicine, 30-1 Oyaguchi-kamicho, Itabashi-ku, Tokyo, 173-8610 Japan

**Keywords:** Fungal endophthalmitis, Corneal transplant, Eye banking, Donor rim cultures, Fortified preservation media, Transplant infection, Diseases, Medical research, Microbiology, Risk factors

## Abstract

This study evaluated the prevalence and microbial spectrum of positive corneoscleral donor rim cultures and the associated risk factors for post-keratoplasty infection in Japan. We retrospectively included consecutive corneal transplantations performed by a single surgeon between April 2015 and September 2021. Donor corneas were obtained from eye banks in Japan (*n* = 134) or the United States (*n* = 365) and preserved using cold storage. Donor corneoscleral rim cultures were obtained during surgery. Culture positivity and microbial profiles were analyzed. Risk factors for contamination were evaluated using logistic regression. Overall, 78 (15.6%) were culture-positive. Domestic grafts had significantly higher microbial contamination rates than overseas grafts (30.6% vs. 10.1%, *P* < 0.001), whereas fungal contamination rates did not differ between groups (1.5% vs. 1.9%, *P* = 1.000). In the multivariate analysis, overseas eye bank origin was independently associated with a lower risk of microbial contamination (OR 0.259, 95% CI, 0.139–0.486; *P* < 0.001). Post-keratoplasty endophthalmitis occurred in three cases (0.6%), all of which were associated with positive fungal cultures. Therefore, fungal pathogens may play a key role in increasing clinically significant postoperative endophthalmitis risk after keratoplasty, highlighting the importance of targeted donor tissue screening and postoperative management.

## Introduction

Corneal transplantation is widely performed as an effective treatment for corneal endothelial disorders, corneal opacity, and hereditary corneal diseases. According to a global survey, more than 180,000 corneal transplantations are performed annually. Recent advances in surgical techniques have led to the development of less invasive corneal transplantation procedures, including deep anterior lamellar keratoplasty (DALK), Descemet’s stripping automated endothelial keratoplasty (DSAEK), Descemet’s membrane endothelial keratoplasty (DMEK), and conventional penetrating keratoplasty (PKP), increasing the number of procedures performed worldwide^1–3^. However, postoperative infectious complications remain a significant concern, with an incidence of infections such as keratitis or endophthalmitis following corneal transplantation known to be higher than that associated with other intraocular surgeries^4,5^. Reports from the Eye Banking Association of America (EBAA) show an increasing trend in infections following keratoplasty^6^. In particular, endophthalmitis, caused by bacterial or fungal infections, often leads to severe visual impairment and poor visual outcomes^7–9^.

Various microorganisms have been reported as causative pathogens of post-keratoplasty infections, including gram-positive cocci, gram-negative bacilli, and fungi such as *Candida*,* Aspergillus*, and *Fusarium*^8,10,11^. Among these, fungal endophthalmitis is particularly difficult to treat and is frequently associated with graft removal, regrafting, and poor visual prognosis^12,13^. In addition, previous studies have demonstrated that donor-related factors play important roles in the development of postoperative infections. Positive donor corneoscleral rim cultures are reported in approximately 7–15% of cases and may be associated with an increased risk of postoperative infection^10^.

Several risk factors for post-keratoplasty infection have been reported, including donor-related factors, preservation conditions of the donor tissue, and the interval between tissue procurement and transplantation. Previous studies have also suggested that contamination of donor corneas with bacteria or fungi may increase the risk of postoperative infection^6,14^.

Despite these findings, it remains unclear whether microbial contamination rates differ between eye bank systems across countries and whether such differences translate into clinically relevant infection risks. Donor procurement and processing workflows differ substantially between Japan and the United States. For example, in Japan, whole-globe retrieval is commonly performed before corneoscleral button preparation, whereas U.S. eye banks generally prepare donor tissue within standardized eye-bank facilities according to EBAA standards. Therefore, this study aimed to compare the prevalence and microbial spectrum of donor corneoscleral rim contamination between Japanese and U.S. eye banks and to evaluate its association with postoperative infection following keratoplasty.

## Methods

### Study design and patients

This study was approved by the Institutional Review Board of Nihon University Hospital (approval no. RK-230411-8), and adhered to the tenets of the Declaration of Helsinki. All patients provided informed consent in the form of opt-outs on the website. Patients who refused to participate were excluded.

The retrospective study population comprised eyes that underwent keratoplasty at Yokohama Minami Kyosai Hospital and the Kikuna Yuda Eye Clinic between April 2015, and September 2021. Surgical procedures included PKP, lamellar keratoplasty (LKP), DALK, DMEK, and DSAEK. All corneal transplantation procedures were performed by a single surgeon (T.H.).

Clinical data were accessed and analyzed by investigators who were formally approved Institutional Review Board members or collaborators between May 1, 2025, and December 31, 2025. Data sharing across institutions was conducted in a fully anonymized manner, and no personally identifiable information was accessible to the investigators outside the institution of origin.

Donor corneas were obtained from domestic eye banks in Japan (Chiba, Saitama, Osaka, Toyama, Gifu, Kanagawa, Gunma, Nagasaki, Nagano, and Shizuoka) and overseas eye banks (CorneaGen, Lions Eye Institute, Mid-America Transplant, and Cincinnati Eye Bank). All donor corneas were preserved in Optisol-GS storage medium (Bausch and Lomb) under standard hypothermic conditions. Pre-cut DMEK and DSAEK tissues supplied by U.S. eye banks had undergone secondary processing and quality-control assessment before shipment according to each eye bank’s standard procedures.

At the time of surgery, a portion of the donor corneoscleral rim was aseptically obtained before transplantation and transferred to a sterile transport container (under anaerobic conditions when required) for microbiological culture.

### Microbiological examination

The collected donor corneal specimens were aseptically divided and inoculated into four types of culture media: CHROMagar Candida Plus agar, Brucella HK agar, sheep blood agar, and chocolate II agar.

For bacterial detection, cultures were incubated aerobically at 35 °C for 72 h. Agar plates were examined at 24-h intervals for colony formation. When bacterial colonies were observed, the organisms were sub-cultured on sheep blood agar for isolation and subsequently identified using matrix-assisted laser desorption/ionization time-of-flight mass spectrometry (MALDI-TOF MS) according to standard laboratory protocols.

For fungal detection, colonies observed on CHROMagar Candida Plus agar were sub-cultured on potato dextrose agar and incubated under appropriate conditions to promote fungal growth. Fungal species were identified based on the colony morphology and microscopic examination using lactophenol cotton blue staining.

Brucella HK agar was used for the detection of anaerobic bacteria under anaerobic conditions. Donor corneas in which bacterial or fungal organisms were detected were defined as culture-positive. Bacterial and fungal contaminations were analyzed separately. All microbiological procedures were performed in accordance with standard clinical microbiology laboratory guidelines.

### Data collection

Clinical variables were collected for both recipients and donors. Recipient variables included age, sex, preoperative diagnosis, surgical procedure, medical history (diabetes mellitus, hypertension, dyslipidemia, and heart disease), and use of anticoagulant medications. Donor variables included donor age, donor sex, cause of death, donor medical history (hypertension, diabetes mellitus, and dyslipidemia), and death-to-preservation interval (preservation time).

### Statistical analysis

The demographic and clinical characteristics of corneal graft donors from domestic and overseas eye banks were compared (Table 1). Continuous variables, including donor age and graft preparation time, were summarized as mean (standard deviation [SD]) and median [range] and compared using the Wilcoxon rank-sum test. Categorical variables, including donor sex, race, cause of death, hypertension, diabetes mellitus, dyslipidemia, microbial culture results, fungal culture results, bacterial culture results, and bacterial species, were summarized as frequencies and percentages and compared using Pearson’s chi-square test. Race categories were based on eye bank–provided classifications.

**Table 1:** Clinical characteristics of corneal graft donors by eye bank location.

Item [unit]	Statistics category	Overall (*N* = 499)	Location of eye bank		Comparison [1] vs. [2]
			[1] Domestic (*N* = 134)	[2] Overseas (*N* = 365)	
Donor age [years]	Mean (SD), N	69.1 (12.9), 499	81.5 (13.6), 134	64.6 (9.1), 365	*P* < 0.001
	Median [range]	68.0 [15, 102]	84.0 [15, 102]	66.0 [21, 101]	
Sex	[1] Male	275 (55.1%)	71 (53.0%)	204 (55.9%)	*P* = 0.670
	[2] Female	222 (44.5%)	62 (46.3%)	160 (43.8%)	
	[3] Unknown	2 (0.4%)	1 (0.7%)	1 (0.3%)	
Donor race	[1] Caucasian	321 (64.3%)	1 (0.7%)	320 (87.7%)	*P* < 0.001
	[2] Asian	142 (28.5%)	133 (99.3%)	9 (2.5%)	
	[3] Black	15 (3.0%)	0 (0.0%)	15 (4.1%)	
	[4] Hispanic	13 (2.6%)	0 (0.0%)	13 (3.6%)	
	[5] Native American	3 (0.6%)	0 (0.0%)	3 (0.8%)	
	[6] Other	1 (0.2%)	0 (0.0%)	1 (0.3%)	
	[7] Unknown	4 (0.8%)	0 (0.0%)	4 (1.1%)	
Cause of death	[1] Cancer	121 (24.2%)	26 (19.4%)	95 (26.0%)	*P* < 0.001
	[2] Heart disease	120 (24.0%)	20 (14.9%)	100 (27.4%)	
	[3] Multiple organ failure	66 (13.2%)	7 (5.2%)	59 (16.2%)	
	[4] Stroke	53 (10.6%)	18 (13.4%)	35 (9.6%)	
	[5] Respiratory failure	52 (10.4%)	12 (9.0%)	40 (11.0%)	
	[6] Infection	45 (9.0%)	27 (20.1%)	18 (4.9%)	
	[7] Old age	21 (4.2%)	21 (15.7%)	0 (0.0%)	
	[8] Trauma	21 (4.2%)	3 (2.2%)	18 (4.9%)	
Hypertension	[1] No	160 (32.1%)	13 (9.7%)	147 (40.3%)	*P* = 0.001
	[2] Yes	210 (42.1%)	2 (1.5%)	208 (57.0%)	
	[3] Unknown	129 (25.9%)	119 (88.8%)	10 (2.7%)	
Diabetes mellitus	[1] No	272 (54.5%)	13 (9.7%)	259 (71.0%)	*P* = 0.379
	[2] Yes	98 (19.6%)	2 (1.5%)	96 (26.3%)	
	[3] Unknown	129 (25.9%)	119 (88.8%)	10 (2.7%)	
Dyslipidemia	[1] No	256 (51.3%)	15 (11.2%)	241 (66.0%)	*P* = 0.019
	[2] Yes	114 (22.8%)	0 (0.0%)	114 (31.2%)	
	[3] Unknown	129 (25.9%)	119 (88.8%)	10 (2.7%)	
Death to preparation [h]	Mean (SD), N	11.01 (5.50), 427	8.99 (5.73), 102	11.64 (5.28), 325	*P* < 0.001
	Median [range]	10.00 [1.5, 23.9]	7.32 [1.5, 23.8]	10.62 [2.8, 23.9]	
Microbial culture	[1] Negative	421 (84.4%)	93 (69.4%)	328 (89.9%)	*P* < 0.001
	[2] Positive	78 (15.6%)	41 (30.6%)	37 (10.1%)	
Fungal culture	[1] Negative	490 (98.2%)	132 (98.5%)	358 (98.1%)	*P* = 1.000
	[2] Positive	9 (1.8%)	2 (1.5%)	7 (1.9%)	
Bacterial culture	[1] Negative	430 (86.2%)	95 (70.9%)	335 (91.8%)	*P* < 0.001
	[2] Positive	69 (13.8%)	39 (29.1%)	30 (8.2%)	
Bacterial species	[1] gpc	53 (10.6%)	31 (23.1%)	22 (6.0%)	*P* = 0.729
	[2] gpr	7 (1.4%)	4 (3.0%)	3 (0.8%)	
	[3] gnr	3 (0.6%)	1 (0.7%)	2 (0.5%)	
	[4] gpc + gnr	2 (0.4%)	1 (0.7%)	1 (0.3%)	
	[5] gnr + gnr	1 (0.2%)	0 (0.0%)	1 (0.3%)	
	[6] gnr + gpr	1 (0.2%)	1 (0.7%)	0 (0.0%)	
	[7] gpc + gpr	1 (0.2%)	1 (0.7%)	0 (0.0%)	

Continuous variables were compared using the Wilcoxon rank-sum test and categorical variables were compared using Pearson’s chi-squared test. Participants with missing or unknown data were excluded from analysis.

gpc, gram-positive cocci; gpr, gram-positive rods; gnr, gram-negative rods; SD, standard deviation.

The clinical characteristics of corneal graft recipients from domestic and overseas eye banks were compared (Table 2). Continuous variables including recipient age were compared using the Wilcoxon rank-sum test. Categorical variables, including recipient sex, laterality, preoperative diagnosis, surgical procedure, glaucoma, diabetes mellitus, hypertension, heart disease, and occurrence of post-keratoplasty endophthalmitis, were compared using Pearson’s chi-squared test.

**Table 2:** Clinical characteristics of corneal graft recipients by eye bank location.

Item [unit]	Statistics category	Overall (*N* = 499)	Location of eye bank		Comparison [1] vs. [2]
			[1] Domestic (*N* = 134)	[2] Overseas (*N* = 365)	
Recipient age [years]	Mean (SD), N	69.8 (14.9), 492	68.0 (17.0), 131	70.4 (14.1), 361	*P* = 0.383
	Median [range]	73.0 [6, 91]	73.0 [6, 89]	74.0 [17, 91]	
Recipient sex	[1] Male	225 (45.1%)	57 (42.5%)	168 (46.0%)	*P* = 0.581
	[2] Female	272 (54.5%)	76 (56.7%)	196 (53.7%)	
	[3] Unknown	2 (0.4%)	1 (0.7%)	1 (0.3%)	
Right or left eye	[1] Right	262 (52.5%)	74 (55.2%)	188 (51.5%)	*P* = 0.510
	[2] Left	234 (46.9%)	59 (44.0%)	175 (47.9%)	
	[3] Unknown	3 (0.6%)	1 (0.7%)	2 (0.5%)	
Diagnosis	[1] BK	225 (45.1%)	16 (11.9%)	209 (57.3%)	*P* < 0.001
	[2] Opacity	107 (21.4%)	47 (35.1%)	60 (16.4%)	
	[3] Failed KP	70 (14.0%)	12 (9.0%)	58 (15.9%)	
	[4] Corneal thinning	66 (13.2%)	52 (38.8%)	14 (3.8%)	
	[5] KC	31 (6.2%)	7 (5.2%)	24 (6.6%)	
Procedure	[1] DMEK	154 (30.9%)	4 (3.0%)	150 (41.1%)	*P* < 0.001
	[2] PKP	125 (25.1%)	49 (36.6%)	76 (20.8%)	
	[3] DALK	91 (18.2%)	37 (27.6%)	54 (14.8%)	
	[4] DSAEK	78 (15.6%)	2 (1.5%)	76 (20.8%)	
	[5] LKP	51 (10.2%)	42 (31.3%)	9 (2.5%)	
Endophthalmitis after transplantation	[1] Negative	496 (99.4%)	134 (100.0%)	362 (99.2%)	*P* = 0.690
	[2] Positive	3 (0.6%)	0 (0.0%)	3 (0.8%)	

Continuous variables were compared using the Wilcoxon rank-sum test and categorical variables were compared using Pearson’s chi-squared test. Participants with missing or unknown data were excluded from analysis.

BK, bullous keratopathy; KP, keratoplasty; KC, keratoconus; DMEK, Descemet’s membrane endothelial keratoplasty; PKP, penetrating keratoplasty; DALK, deep anterior lamellar keratoplasty; DSAEK, Descemet’s stripping automated endothelial keratoplasty; LKP, lamellar keratoplasty; SD, standard deviation.

Donor and recipient characteristics were compared between patients with and without post-keratoplasty endophthalmitis (Table 3). Given the small number of endophthalmitis cases, continuous variables were compared using the Wilcoxon rank-sum test and categorical variables were compared using Fisher’s exact test.

**Table 3:** Clinical characteristics of donors and recipients with or without post-keratoplasty endophthalmitis.

Item [unit]	Statistics category	Post-keratoplasty endophthalmitis		Comparison [1] vs. [2]
		[1] Negative (*N* = 496)	[2] Positive (*N* = 3)	
Eye bank location	[1] Domestic	134 (27.0%)	0 (0.0%)	*P* = 0.568
	[2] Overseas	362 (73.0%)	3 (100.0%)	
Donor age [years]	Mean (SD), N	69.2 (12.9), 496	56.0 (1.0), 3	*P* = 0.017
	Median [range]	69.0 [15, 102]	56.0 [55, 57]	
Donor sex	[1] Male	274 (55.2%)	1 (33.3%)	*P* = 0.589
	[2] Female	220 (44.4%)	2 (66.7%)	
	[3] Unknown	2 (0.4%)	0 (0.0%)	
Donor race	[1] Caucasian	318 (64.1%)	3 (100.0%)	*P* = 0.625
	[2] Asian	142 (28.6%)	0 (0.0%)	
	[3] Black	15 (3.0%)	0 (0.0%)	
	[4] Hispanic	13 (2.6%)	0 (0.0%)	
	[5] Native American	3 (0.6%)	0 (0.0%)	
	[6] Other	1 (0.2%)	0 (0.0%)	
	[7] Unknown	4 (0.8%)	0 (0.0%)	
Donor cause of death	[1] Cancer	121 (24.4%)	0 (0.0%)	*P* = 0.025
	[2] Heart disease	120 (24.2%)	0 (0.0%)	
	[3] Multiple organ failure	64 (12.9%)	2 (66.7%)	
	[4] Stroke	53 (10.7%)	0 (0.0%)	
	[5] Respiratory failure	52 (10.5%)	0 (0.0%)	
	[6] Infection	45 (9.1%)	0 (0.0%)	
	[7] Old age	21 (4.2%)	0 (0.0%)	
	[8] Trauma	20 (4.0%)	1 (33.3%)	
Donor hypertension	[1] No	158 (31.9%)	2 (66.7%)	*P* = 0.581
	[2] Yes	209 (42.1%)	1 (33.3%)	
	[3] Unknown	129 (26.0%)	0 (0.0%)	
Donor diabetes mellitus	[1] No	269 (54.2%)	3 (100.0%)	*P* = 0.569
	[2] Yes	98 (19.8%)	0 (0.0%)	
	[3] Unknown	129 (26.0%)	0 (0.0%)	
Donor dyslipidemia	[1] No	253 (51.0%)	3 (100.0%)	*P* = 0.556
	[2] Yes	114 (23.0%)	0 (0.0%)	
	[3] Unknown	129 (26.0%)	0 (0.0%)	
Death to preparation [h]	Mean (SD), N	11.01 (5.51), 426	8.55 (NA), 1	*P* = 0.761
	Median [range]	10.03 [1.5, 23.9]	8.55 [8.6, 8.6]	
Microbial culture	[1] Negative	421 (84.9%)	0 (0.0%)	*P* = 0.004
	[2] Positive	75 (15.1%)	3 (100.0%)	
Fungal culture	[1] Negative	490 (98.8%)	0 (0.0%)	*P* < 0.001
	[2] Positive	6 (1.2%)	3 (100.0%)	
Bacterial culture	[1] Negative	427 (86.1%)	3 (100.0%)	*P* = 1.000
	[2] Positive	69 (13.9%)	0 (0.0%)	
Recipient, Age [years]	Mean (SD), N	69.7 (15.0), 489	81.7 (1.5), 3	*P* = 0.049
	Median [range]	73.0 [6, 91]	82.0 [80, 83]	
Recipient, Sex	[1] Male	225 (45.4%)	0 (0.0%)	*P* = 0.255
	[2] Female	269 (54.2%)	3 (100.0%)	
	[3] Unknown	2 (0.4%)	0 (0.0%)	
Right or left eye	[1] Right	262 (52.8%)	0 (0.0%)	*P* = 0.104
	[2] Left	231 (46.6%)	3 (100.0%)	
	[3] Unknown	3 (0.6%)	0 (0.0%)	
Diagnosis	[1] BK	222 (44.8%)	3 (100.0%)	*P* = 0.874
	[2] Opacity	107 (21.6%)	0 (0.0%)	
	[3] Failed KP	70 (14.1%)	0 (0.0%)	
	[4] Corneal thinning	66 (13.3%)	0 (0.0%)	
	[5] KC	31 (6.2%)	0 (0.0%)	
Procedure	[1] DMEK	153 (30.8%)	1 (33.3%)	*P* = 0.171
	[2] PKP	125 (25.2%)	0 (0.0%)	
	[3] DALK	91 (18.3%)	0 (0.0%)	
	[4] DSAEK	76 (15.3%)	2 (66.7%)	
	[5] LKP	51 (10.3%)	0 (0.0%)	
Recipient glaucoma	[1] No	489 (98.6%)	3 (100.0%)	*P* = 1.000
	[2] Yes	2 (0.4%)	0 (0.0%)	
	[3] Unknown	5 (1.0%)	0 (0.0%)	
Recipient diabetes mellitus	[1] No	466 (94.0%)	3 (100.0%)	*P* = 1.000
	[2] Yes	26 (5.2%)	0 (0.0%)	
	[3] Unknown	4 (0.8%)	0 (0.0%)	
Recipient hypertension	[1] No	488 (98.4%)	3 (100.0%)	*P* = 1.000
	[2] Yes	4 (0.8%)	0 (0.0%)	
	[3] Unknown	4 (0.8%)	0 (0.0%)	
Recipient heart disease	[1] No	476 (96.0%)	3 (100.0%)	*P* = 1.000
	[2] Yes	9 (1.8%)	0 (0.0%)	
	[3] Unknown	11 (2.2%)	0 (0.0%)	

Continuous variables were compared using the Wilcoxon rank-sum test and categorical variables were compared using Fisher’s exact test. Participants with missing or unknown data were excluded from analysis.

BK, bullous keratopathy; KP, keratoplasty; KC, keratoconus; DMEK, Descemet’s membrane endothelial keratoplasty; PKP, penetrating keratoplasty; DALK, deep anterior lamellar keratoplasty; DSAEK, Descemet’s stripping automated endothelial keratoplasty; LKP, lamellar keratoplasty; SD, standard deviation.

Donor characteristics were stratified according to microbial graft culture results (positive vs. negative) to identify potential factors associated with graft contamination (Table 4). Continuous variables were compared using the Wilcoxon rank-sum test and categorical variables were compared using Pearson’s chi-squared test.

**Table 4:** Clinical characteristics of donors stratified by microbial graft culture results.

Item [unit]	Statistics category	Microbial graft culture		Comparison [1] vs. [2]
		[1] Negative (*N* = 421)	[2] Positive (*N* = 78)	
Eye bank location	[1] Domestic	93 (22.1%)	41 (52.6%)	*P* < 0.001
	[2] Overseas	328 (77.9%)	37 (47.4%)	
Donor age [years]	Mean (SD), N	68.3 (12.2), 421	73.6 (15.7), 78	*P* < 0.001
	Median [range]	68.0 [16, 102]	74.5 [15, 97]	
Donor sex	[1] Male	238 (56.5%)	37 (47.4%)	*P* = 0.160
	[2] Female	181 (43.0%)	41 (52.6%)	
	[3] Unknown	2 (0.5%)	0 (0.0%)	
Donor race	[1] Caucasian	290 (68.9%)	31 (39.7%)	*P* < 0.001
	[2] Asian	99 (23.5%)	43 (55.1%)	
	[3] Black	15 (3.6%)	0 (0.0%)	
	[4] Hispanic	10 (2.4%)	3 (3.8%)	
	[5] Native American	3 (0.7%)	0 (0.0%)	
	[6] Other	1 (0.2%)	0 (0.0%)	
	[7] Unknown	3 (0.7%)	1 (1.3%)	
Donor cause of death	[1] Cancer	102 (24.2%)	19 (24.4%)	*P* = 0.958
	[2] Heart disease	102 (24.2%)	18 (23.1%)	
	[3] Multiple organ failure	57 (13.5%)	9 (11.5%)	
	[4] Stroke	45 (10.7%)	8 (10.3%)	
	[5] Respiratory failure	42 (10.0%)	10 (12.8%)	
	[6] Infection	39 (9.3%)	6 (7.7%)	
	[7] Old age	16 (3.8%)	5 (6.4%)	
	[8] Trauma	18 (4.3%)	3 (3.8%)	
Donor hypertension	[1] No	143 (34.0%)	17 (21.8%)	*P* = 0.981
	[2] Yes	189 (44.9%)	21 (26.9%)	
	[3] Unknown	89 (21.1%)	40 (51.3%)	
Donor diabetes mellitus	[1] No	241 (57.2%)	31 (39.7%)	*P* = 0.320
	[2] Yes	91 (21.6%)	7 (9.0%)	
	[3] Unknown	89 (21.1%)	40 (51.3%)	
Donor dyslipidemia	[1] No	229 (54.4%)	27 (34.6%)	*P* = 0.938
	[2] Yes	103 (24.5%)	11 (14.1%)	
	[3] Unknown	89 (21.1%)	40 (51.3%)	
Death to preparation [h]	Mean (SD), N	11.06 (5.42), 365	10.68 (5.98), 62	*P* = 0.496
	Median [range]	10.05 [1.6, 23.9]	8.97 [1.5, 23.6]	

Continuous variables were compared using the Wilcoxon rank-sum test and categorical variables were compared using Pearson’s chi-squared test. Participants with missing or unknown data were excluded from analysis. SD, standard deviation.

Risk factors for a positive microbial graft culture (Table 5) was evaluated using logistic regression. Three explanatory variables were considered: eye bank location^1^(Domestic, Overseas), donor age (modeled per 10-year increment), and surgical procedure (DMEK; DMEK, Descemet membrane endothelial keratoplasty; PKP, penetrating keratoplasty; DALK, deep anterior lamellar keratoplasty; DSAEK, Descemet stripping automated endothelial keratoplasty; LKP, lamellar keratoplasty). For each outcome, univariable models were fitted for each explanatory variable separately, and a multivariable model was fitted including all three variables simultaneously. Odds ratios with 95% confidence intervals (CIs) were estimated for each model.

**Table 5:** Risk factor analysis for positive microbial graft culture results using logistic regression model (complete-case, *N* = 499).

Explanatory variable	Category Unit	Univariable model				Multivariable model		
		Odds ratio	95% CI	*P* value		Odds ratio	95% CI	*P* value
Eye bank location	[1] Domestic	Reference	-	< 0.001		Reference	-	0.003
	[2] Overseas	0.256	[0.155, 0.422]			0.347	[0.170, 0.703]	
Donor age	10 years	1.385	[1.143, 1.686]	0.001		1.017	[0.817, 1.285]	0.879
Procedure	[1] DMEK	Reference	-	< 0.001		Reference	-	0.325
	[2] PKP	2.887	[1.468, 5.912]			1.830	[0.859, 3.979]	
	[3] DALK	2.466	[1.165, 5.319]			1.517	[0.661, 3.483]	
	[4] DSAEK	0.685	[0.215, 1.868]			0.687	[0.215, 1.884]	
	[5] LKP	3.421	[1.472, 7.939]			1.89	[0.510, 3.737]	

OR, odds ratio; CI, confidence interval. Model specification, the variable-wise likelihood-ratio P value shown on each reference row, and the complete-case analysis are described in the Statistical methods section.

For the categorical variables (eye bank location and surgical procedure), the overall significance of each variable was assessed with a likelihood-ratio (ANOVA-type) test for the variable as a whole; this single P value is presented on the reference-category row of the tables, and the individual category rows are intentionally left blank. For donor age (continuous), the P value is the Wald test for its regression coefficient. Subjects with a missing value in any model variable were excluded (complete-case analysis); no exclusions occurred, so all 499 grafts were analyzed in both models. A two-sided *P* < 0.05 was considered statistically significant. All analyses were performed in R (R version 4.6.0 (2026-04-24)).

## Results

### Donor characteristics by eye bank location

Among 499 corneal grafts included in this study, 134 (26.9%) were obtained from domestic eye banks and 365 (73.1%) from overseas eye banks (Table 1). The mean donor age was significantly higher in the domestic group (81.5 ± 13.6 years) compared with the overseas group (64.6 ± 9.1 years; *P* < 0.001). There was no significant difference in the donor sex between the two groups (*P* = 0.670). The racial composition differed significantly between the groups; domestic donors were predominantly Asian (99.3%), whereas overseas donors were predominantly Caucasian (87.7%; *P* < 0.001). The distribution of causes of death also differed significantly (*P* < 0.001), with infection (20.1%) and old age (15.7%) being more common among domestic donors, whereas heart disease (27.4%) and cancer (26.0%) were more prevalent among overseas donors. Among donors with available comorbidity data, hypertension (*P* = 0.001) and dyslipidemia (*P* = 0.019) were significantly more common in the overseas group, whereas diabetes mellitus showed no significant difference (*P* = 0.379). The mean graft preparation time was significantly shorter for domestic grafts (8.99 ± 5.73 h) than for overseas grafts (11.64 ± 5.28 h; *P* < 0.001). The overall rate of positive microbial cultures was 15.6%, which was significantly higher in the domestic group (30.6%) than that in the overseas group (10.1%; *P* < 0.001). Similarly, the rate of positive bacterial cultures was significantly higher in the domestic group (29.1% vs. 8.2%, *P* < 0.001). The rate of positive fungal cultures was low in both groups (1.5% vs. 1.9%), with no significant difference (*P* = 1.000). Gram-positive cocci were the most frequently isolated bacterial culture-positive grafts (53/69, 76.8%).

## Recipient characteristics by eye bank location

The clinical characteristics of the 499 corneal graft recipients are shown in Table 2. There were no significant differences between the domestic and overseas groups in recipient age (mean 68.0 ± 17.0 vs. 70.4 ± 14.1 years; *P* = 0.383), sex (*P* = 0.581), or laterality (*P* = 0.510). The distribution of preoperative diagnoses differed significantly between the groups (*P* < 0.001); bullous keratopathy (BK) was the most common indication in the overseas group (57.3%), whereas corneal thinning (38.8%) and opacity (35.1%) were more prevalent in the domestic group. Surgical procedures also differed significantly (*P* < 0.001); DMEK was the most common procedure in the overseas group (41.1%), whereas PKP (36.6%), DALK (27.6%), and LKP (31.3%) were predominant in the domestic group. There were no significant differences in the prevalence of glaucoma (*P* = 0.961), diabetes mellitus (*P* = 0.843), hypertension (*P* = 0.623), and heart disease (*P* = 0.960) between the two groups. Post-keratoplasty endophthalmitis occurred in 3 cases (0.6%), all in recipients of overseas grafts, with no significant difference between the groups (*P* = 0.690; Table 3).

## Characteristics associated with post-keratoplasty endophthalmitis

The donors of the three grafts associated with post-keratoplasty endophthalmitis were significantly younger (mean 56.0 ± 1.0 years) than those without endophthalmitis (mean 69.2 ± 12.9 years; *P* = 0.017). Among the donors of endophthalmitis-associated grafts, two (66.7%) died of multiple organ failure and one (33.3%) died from trauma, with a significant difference in the distribution of causes of death compared with the non-endophthalmitis group (*P* = 0.025). All three endophthalmitis cases had positive microbial graft cultures (100.0% vs. 15.1%; *P* = 0.004) and all three had positive fungal graft cultures (100.0% vs. 1.2%; *P* < 0.001), whereas none had positive bacterial cultures. Recipients who developed endophthalmitis were significantly older (mean 81.7 ± 1.5 years vs. 69.7 ± 15.0 years; *P* = 0.049). All three affected recipients were female, and underwent surgery in the left eye. All three patients were diagnosed with BK; two underwent DSAEK, and one underwent DMEK. One patient was treated with a single injection of an antifungal drug (amphotericin B). Another patient required transplanted donor tissue removal as well as the injection of an antifungal drug (amphotericin B). The severe case was treated with pars plana vitrectomy and irrigation with the antifungal drug amphotericin B.

### Donor characteristics by microbial graft culture results

Among 499 corneal grafts, 78 (15.6%) were positive for microbial graft cultures (Table 4). Grafts from domestic eye banks had a significantly higher rate of positive microbial cultures (52.6%) than the overall proportion of domestic grafts (*P* < 0.001). Donors with positive microbial cultures were significantly older (mean 73.6 ± 15.7 years vs. 68.3 ± 12.2 years; *P* < 0.001). Racial distribution differed significantly between the culture-positive and culture-negative groups (*P* < 0.001), with Asian donors comprising 55.1% and 23.5% of the positive and negative groups, respectively. There were no significant differences in donor sex (*P* = 0.160), cause of death (*P* = 0.958), hypertension (*P* = 0.981), diabetes mellitus (*P* = 0.320), dyslipidemia (*P* = 0.938), or graft preparation time (*P* = 0.496) between the two groups.

### Donor characteristics by fungal graft culture results

Only 9 of the 499 grafts (1.8%) had positive fungal cultures. There were no significant differences between the fungal culture-positive and culture-negative groups in terms of eye bank location (*P* = 1.000), donor age (*P* = 0.339), donor sex (*P* = 0.093), race (*P* = 0.966), cause of death (*P* = 0.075), hypertension (*P* = 0.453), diabetes mellitus (*P* = 0.616), dyslipidemia (*P* = 0.455), or graft preparation time (*P* = 0.230). Among the nine fungal culture-positive patients, seven (77.8%) were female, and multiple organ failure was the most common cause of death (4/9, 44.4%).

### Donor characteristics by bacterial graft culture results

Among the 499 grafts, 69 (13.8%) had positive bacterial cultures. Grafts from domestic eye banks were significantly overrepresented in the positive group (56.5% domestic vs. 22.1% negative group; *P* < 0.001). Donors with positive bacterial cultures were significantly older (mean 74.7 ± 15.5 years vs. 68.2 ± 12.2 years; *P* < 0.001), and the racial distribution differed significantly (*P* < 0.001), with Asian donors comprising 59.4% of the positive group compared with 23.5% of the negative group. There were no significant differences in terms of donor sex (*P* = 0.484), cause of death (*P* = 0.784), hypertension (*P* = 0.856), diabetes mellitus (*P* = 0.533), dyslipidemia (*P* = 0.916), or graft preparation time (*P* = 0.771) between the two groups.

### Risk factor analysis for graft culture positivity

Logistic regression analysis for microbial graft culture positivity revealed that overseas eye bank origin was significantly associated with a lower risk of positive microbial culture in both univariate (OR = 0.256; 95% CI: 0.155–0.422; *P* < 0.001) and multivariate (OR = 0.259; 95% CI: 0.139–0.486; *P* < 0.001) models. Donor age (per 10-year increment) was a significant risk factor in the univariate model (OR = 1.385; 95% CI: 1.143–1.686; *P* = 0.001), but lost significance after adjusting for eye bank location in the multivariable model (OR = 1.007; 95% CI: 0.812–1.266; *P* = 0.952).

A positive microbial graft culture was observed in 78 of 499 grafts. In univariable analyses, an overseas eye bank was associated with significantly lower odds of a positive microbial culture (odds ratio [OR] 0.256, 95% CI [0.155, 0.422]; *P* < 0.001), older donor age was associated with higher odds (OR 1.385 per 10 years, 95% CI [1.143, 1.686]; *P* = 0.001), and surgical procedure was significant overall (*P* < 0.001). In the multivariable model, an overseas eye bank remained independently associated with lower odds (OR 0.347, 95% CI [0.170, 0.703]; *P* = 0.003), whereas donor age (OR 1.017 per 10 years; *P* = 0.879) and surgical procedure (overall *P* = 0.325) were no longer significant. (Table 5)

In the multivariable model, an overseas eye bank remained independently associated with lower odds of a positive bacterial culture (OR 0.359, 95% CI [0.171, 0.752]; *P* = 0.007), and surgical procedure remained significant overall (overall *P* = 0.039), whereas donor age was not (OR 1.066 per 10 years; *P* = 0.603). Among the procedure categories, PKP had the largest point estimate relative to DMEK (OR 2.256, 95% CI [1.009, 5.243]); however, this was an exploratory, post-hoc pairwise contrast with a confidence interval that only marginally excluded 1, and should therefore be interpreted cautiously.

## Discussion

In this study, donor corneas obtained from domestic eye banks were significantly more likely to have positive microbial cultures than those obtained from overseas eye banks. The most frequently detected organisms were bacterial species, particularly gram-positive cocci such as *Staphylococcus epidermidis*. Because the preservation medium used for donor corneas was identical (Optisol-GS) in both domestic and overseas donors, differences in storage media were unlikely to explain this finding.

One possible explanation may be related to differences in donor characteristics. In Japan, donors tend to be older and older individuals are generally more likely to receive antimicrobial therapy during hospitalization, particularly in the presence of multiple comorbidities^14,15^. Such exposure could theoretically influence the microbial flora of donor tissues. However, in the present study, although donor age was associated with microbial contamination in the univariate analysis, this association was no longer significant after adjusting for eye bank location in the multivariate model. These findings suggest that donor age alone is unlikely to account for the higher contamination rates observed in domestic donor corneas. Additionally, antimicrobial exposure was not directly assessed in this study; therefore, this hypothesis remains speculative and should be interpreted with caution.

Another possible explanation is related to differences in eye-bank systems and tissue procurement practices^1,6^. In the United States, eye bank technicians are generally required to obtain certification through the Eye Bank Association of America (EBAA), which includes competency assessment under the supervision of a Medical Director. In addition, continuing education and recertification every three years are required. The EBAA Medical Standards also require ongoing competency assessment, including annual performance evaluations and documentation of when personnel are deemed qualified to perform procedures independently^6^.

In contrast, donor tissue procurement in Japan is primarily managed by regional eye banks, and no nationwide certification system equivalent to the Certified Eye Banker Technician (CEBT) program currently exists. Because ocular tissue recovery is considered a medical procedure, enucleation is generally performed by ophthalmologists. However, there are no nationally standardized requirements regarding years of clinical experience, certification examinations, or periodic recertification for personnel involved in donor tissue recovery.

In addition, tissue procurement workflows differ between the two countries. In Japan, donor eyes are commonly recovered as whole globes and transported to an eye bank or affiliated facility before corneoscleral button preparation, whereas in the United States, corneoscleral tissue is often recovered directly at the donor site before transportation to the eye bank. Consequently, the interval between tissue recovery and placement into Optisol-GS may be shorter in the United States. Furthermore, unlike the United States, where the Eye Bank Association of America (EBAA) provides a standardized certification, competency assessment, and recertification system for eye bank personnel, Japan currently lacks a nationwide certification program or unified training framework specifically for donor tissue recovery. Ocular tissue procurement is generally performed by ophthalmologists affiliated with regional eye banks, often in addition to their routine clinical duties. Consequently, personnel experience, recovery techniques, and quality-control practices may vary among institutions. Although the impact of these differences could not be directly assessed in the present study, the absence of a standardized national training and certification system may contribute to inter-institutional variability in donor tissue handling and preservation procedures. These differences in personnel training, quality-control systems, tissue recovery workflows, and preservation practices may potentially influence microbial contamination rates^1,16^. Although these factors were not directly evaluated in the present study, they may have partially contributed to the higher rate of bacterial contamination observed in the corneas of domestic donors^10,16^. Since all imported tissues were supplied by EBAA-accredited eye banks, the lower contamination rate observed in overseas grafts may partly reflect the benefits of standardized procurement, tissue handling, and quality-control procedures. standardization of tissue procurement and preservation protocols, along with the development of effective strategies to prevent fungal contamination, may improve the safety of corneal transplantation.

Interestingly, the rate of fungal contamination did not differ significantly between domestic and overseas donor corneas. Commonly used corneal preservation media, including Optisol-GS, contain antibacterial agents, but lack antifungal components^17^. If differences in contamination were primarily due to variations in procurement techniques or handling conditions, both bacterial and fungal contamination rates might differ between the groups. However, this was not observed in this study. In addition, the relatively low number of fungus-positive cases in this study may have limited the statistical power to detect differences between the groups. Therefore, the findings should be interpreted with caution.

**Fig. 1:**
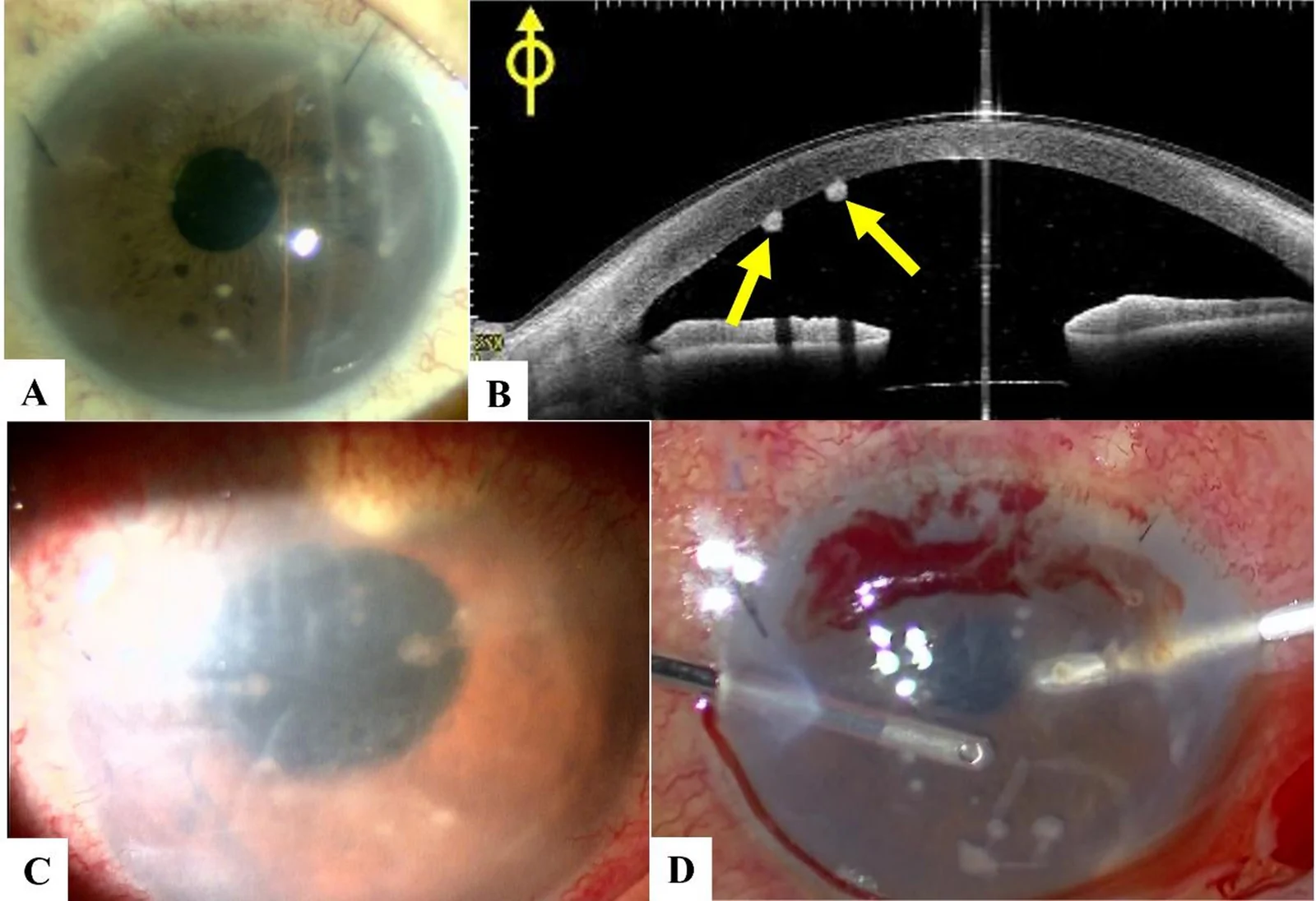
A representative case of donor-related fungal infection. (**a**) A slit lamp photograph indicating an onset of interface fungal infection 17 days after DMEK. (**b**) Anterior segment optical coherence tomography showing infiltration to the posterior surface, indicating fungal infection (arrows). Furthermore, *Candida albicans* was detected by a microbiological test. (**c**) Significant deterioration 1 month post-DMEK, with a substantial amount of hypopyon. (**d**) Following DMEK graft removal, anterior chamber irrigation with antifungal drug.

In our cohort, postoperative endophthalmitis was rare; however, all cases occurred in grafts with positive fungal cultures, and the causative organisms matched those identified in the donor corneal cultures, which is consistent with previous clinical reports^6,10,18^. Notably, all endophthalmitis cases occurred after endothelial keratoplasty procedures using imported donor tissue. Previous EBAA reports have suggested that fungal infections occur more frequently after endothelial keratoplasty than after penetrating keratoplasty, possibly owing to additional tissue manipulation and warming cycles during graft preparation. Fungal endophthalmitis is associated with poor visual outcomes. Previous studies have reported that approximately 6.7% of corneas with positive fungal cultures develop postoperative fungal endophthalmitis^10,18^. Once a fungal infection occurs, the clinical course can be devastating, as illustrated in Fig. 1. However, the number of endophthalmitis cases in this study was small and definitive conclusions regarding causality could not be drawn.

The use of povidone-iodine during tissue procurement has been proposed to reduce microbial contamination, with increased exposure time potentially reducing the rate of positive fungal donor rim cultures^19^. In addition, antimicrobial effects have been reported even at low concentrations of povidone-iodine^20,21^. However, concerns remain regarding their potential toxicity to the corneal endothelial cells. While some studies have suggested that low concentrations of iodine are safe, others have reported endothelial damage even at low concentrations, and no consensus has been established^22–24^. These conflicting findings highlight the need for careful optimization of both concentration and exposure time in clinical practice.

More recently, antifungal supplementation of corneal preservation media was investigated as an alternative strategy. Several studies have reported that the addition of antifungal agents such as amphotericin B significantly reduces fungal contamination rates^10,17,25^. However, concerns remain regarding the potential toxicity to donor endothelial cells, optimal drug concentration, cost-effectiveness, and actual impact on postoperative infection rates^10,25,26^. Furthermore, the resistance profiles of the isolated microorganisms were not evaluated in the present study; therefore, the potential role of antimicrobial-resistant organisms remains unclear. Further investigation is required to determine the clinical utility and safety of antifungal supplementation in corneal preservation media, especially as not all microbial contaminants appear to have equal clinical significance.

This study has several limitations. First, this was a retrospective study conducted at a single institution, which may limit generalizability. Second, microbiological evaluation was based on donor corneoscleral rim cultures, which may not fully reflect the true microbial burden within the donor tissue, resulting in false-negative results. Third, important factors, such as detailed preservation conditions, transportation time, and perioperative antimicrobial prophylaxis, were not fully assessed. Fourth, a substantial proportion of donor clinical variables, particularly comorbidities, were categorized as “unknown,” particularly in the domestic cohort.

Surgical procedures differed substantially between groups, with DMEK and DSAEK predominating in the overseas cohort and PKP, DALK, and LKP predominating in the domestic cohort. Although donor rim cultures were obtained before implantation and therefore were unlikely to be directly influenced by surgical procedure, this imbalance may have affected the observed rates of postoperative infection. Information regarding Optisol-GS lot numbers and preservation records was unavailable for a proportion of domestic donor tissues. Therefore, the potential impact of preservation-medium lots and storage conditions on microbial contamination rates or postoperative endophthalmitis could not be evaluated. In addition, donor endothelial cell density data were unavailable for a substantial proportion of cases because some source records were no longer accessible owing to institutional record-retention policies and the long study period. Consequently, the analysis was limited to variables incorporated into the study database, and the influence of donor endothelial status could not be assessed. Detailed information regarding tissue-recovery protocols at individual U.S. eye banks, including the implementation of infection-control measures such as double povidone-iodine treatment, was also unavailable. Likewise, detailed information regarding tissue-processing methods, warming duration, and quality-control procedures associated with DMEK and DSAEK graft preparation could not be obtained. Therefore, the potential effects of these factors on culture positivity rates and postoperative infectious outcomes could not be evaluated. Furthermore, the number of endophthalmitis cases was small, and no significant difference in fungal culture positivity was observed between domestic and overseas donor tissues. Consequently, the influence of inter-eye-bank procedural differences and tissue-processing practices on postoperative endophthalmitis remains uncertain and warrants further investigation.

This likely reflects differences in data availability and documentation practices between eye bank systems, rather than random missingness. Although such incomplete data may have limited the ability to fully evaluate certain donor-related risk factors, key findings, including the association between eye bank location and microbial contamination, remained robust in the multivariable analysis. Therefore, the impact of missing data on the primary conclusions of this study was limited. This marked imbalance in data availability between the groups should be considered when interpreting donor comorbidity data. Finally, the number of postoperative endophthalmitis cases was small, limiting the statistical power to evaluate associations with clinical outcomes.

In conclusion, the higher bacterial contamination rate observed in Japanese donor corneas may reflect differences in donor procurement and tissue-processing workflows between eye-bank systems. In contrast, endophthalmitis was exclusively associated with fungal contamination, suggesting that fungal pathogens may play a key role in clinically significant infections after keratoplasty.

## Data Availability

The datasets generated and/or analyzed during the current study are available from the corresponding author on reasonable request.
